# Improving Outcomes for Infants with Single Ventricle Physiology through Standardized Feeding during the Interstage

**DOI:** 10.1155/2016/9505629

**Published:** 2016-05-22

**Authors:** Cindy Weston, S. Adil Husain, Christopher L. Curzon, Steve Neish, Gemma T. Kennedy, Krista Bonagurio, Kevin Gosselin

**Affiliations:** ^1^Texas A&M University Health Science Center, College of Nursing, Bryan-College Station, TX, USA; ^2^Department of Cardiothoracic Surgery, University of Texas Health Science Center at San Antonio, San Antonio, TX, USA; ^3^Department of Pediatrics, University of Nebraska, Division of Pediatric Cardiology, Lincoln, NE, USA; ^4^Department of Pediatrics, University of Texas Health Science Center at San Antonio, San Antonio, TX, USA; ^5^Department of Family and Community Health Systems, University of Texas Health Science Center at San Antonio, San Antonio, TX, USA; ^6^University Health System, San Antonio, TX, USA

## Abstract

Congenital heart disease is identified as the most common birth defect with single ventricle physiology carrying the highest mortality. Staged surgical palliation is required for treatment, with mortality historically as high as 22% in the four- to six-month period from the first- to second-stage surgical palliation, known as the interstage. A standardized postoperative feeding approach was implemented through an evidence-based protocol, parent engagement, and interprofessional team rounds. Five infants with single ventricle physiology preprotocol were compared with five infants who received the standardized feeding approach. Mann-Whitney *U* tests were conducted to evaluate the hypotheses that infants in the intervention condition would consume more calories and have a positive change in weight-to-age *z*-score (WAZ) and shorter length of stay (LOS) following the first and second surgeries compared to infants in the control condition. After the protocol, the change in WAZ during the interstage increased by virtually one standard deviation from 0.05 to 0.91. Median LOS dropped 32% after the first surgery and 43% after the second surgery. Since first- and second-stage palliative surgeries occur within the same year of life, this represents savings of $500,000 to $800,000 per year in a 10-infant model. The standardized feeding approach improved growth in single ventricle infants while concurrently lowering hospital costs.

## 1. Introduction

Congenital heart disease is the most common birth defect, occurring in nine out of every 1000 live births, with congenital anomalies being the leading cause of death in infants below one year of age [1]. Many infants with congenital heart disease experience failure to thrive related to impaired nutritional consumption and feeding difficulties. Of all forms of congenital heart disease, those characterized by having single ventricle physiology carry the highest mortality and greatest challenges. For survival, surgery is required in the first days of life further complicating feeding and growth problems.

Typically, three surgical palliations are required in the first few years of life to achieve survival for children born with a single functional ventricle. The goal of the first surgery is to establish a reliable pathway for systemic circulation, control pulmonary circulation, and limit ventricular workload. Depending on the native anatomy, variable approaches are required for the first-stage surgery often involving complete ventricular outflow tract reconstruction and a systemic to pulmonary artery shunt. Usually, the first surgery is performed in the first few days of life. The second palliation classically is performed at four to six months of age and is the first step in separating the systemic circulation from the pulmonary circulation. At this operation, the superior vena cava is anastomosed to the pulmonary artery, and the pulmonary artery is disconnected from the heart. Pulmonary blood flow occurs without being pumped by a ventricle and depends on unobstructed pulmonary arteries and healthy pulmonary microvasculature. This operation, known as the bidirectional Glenn, also decreases workload on the functional single ventricle. The third operation directs the inferior vena cava and hepatic venous return around the heart to the pulmonary arteries. This results in what has come to be known as Fontan circulation, where systemic circulation and pulmonary circulation occur in series and ventricular workload is reduced to support only the systemic circulation. The third-stage palliative surgery occurs around two to four years. With Fontan circulation, all caval blood flow is directed to the pulmonary system while the functional single ventricle is responsible for systemic circulation. The highest mortality, up to 22%, remains in the four- to six-month period from first-stage until second-stage surgical palliation, known as the interstage [2].

Recent advances in these staged palliative procedures have improved survival and according to Lowry [3] seventy percent of these infants are expected to reach adulthood. Although the majority of these children now reach adulthood, many have neurodevelopmental delays attributed to a variety of factors including poor nutrition [4]. Poor nutrition in infants born with single ventricle physiology is related to inherent feeding and growth difficulties. Infants with heart disease struggle with the physical endurance and coordination necessary to suck, swallow, and breathe during feeding in order to balance the consumption and expenditure of calories to provide appropriate nutrition for somatic growth. At the project institution, former practice promoted healthcare provider preference and variability which created inconsistent practices in the timing for the initiation of postoperative feeding, the rate of progression with caloric intake, and the involvement of interprofessional disciplines such as speech therapy and a registered dietitian. In addition, little continuity existed between the inpatient and outpatient growth monitoring and feeding practices for infants during the interstage of single ventricle palliation. In 2012, the Joint Council on Congenital Heart Disease, National Pediatric Cardiology Quality Improvement Collaborative (JCCHD-NPCQIC), reported positive infant weight gain at centers that employed a standardized postoperative feeding evaluation and approach [5]. Parent engagement through education and home monitoring of growth indicators has been recommended as an essential component in standardizing the interstage feeding regimen. This paper reports on a quality improvement project that focused on implementation of this evidence and applies cost savings to the intervention through decreased hospital length of stay.

## 2. Literature Review

For years, healthcare professionals considered infants with single ventricle physiology in the interstage incapable of somatic growth; however, improved growth has been realized with standardized feeding approaches [2, 5–11]. Although no single feeding protocol has been reported to demonstrate superior outcome over others, the process of* standardization*, itself, has emerged as the element that decreases provider variability and improves growth objectives in these vulnerable infants [5]. Higher weight-to-age *z*-scores (WAZ) indicate improved growth. WAZ is the method of central tendency describing body weight mean, frequency distribution, and standard deviation based on the World Health Organization standard for infant gender and age (http://www.who.int/).

Most studies report infants with single ventricle physiology as having a high incidence of failure to thrive which is associated with increased mortality at second-stage palliation, along with increased postoperative complications, poorer long-term neurological outcomes, and longer lengths of hospital stay [6, 7]. Multifactorial causes contribute to poor somatic growth in the interstage including high metabolic demands, inadequate caloric intake, fatiguing with feeds causing poor intake, and gastrointestinal perfusion impairment that results in decreased absorption, along with genetic and extracardiac anomalies [2]. A high incidence of necrotizing enterocolitis (NEC), up to 18%, is associated with enteral feeding of infants with single ventricle physiology which challenges healthcare providers regarding pace of advancing nutrition [12]. NEC does not only contribute to increased morbidity but a mortality of 38% is seen in infants with single ventricle physiology who develop NEC [12]. Modifiable contributors to improve WAZ center on optimizing calories through a standardized feeding approach which leads to improved growth outcomes during the interstage, lower mortality, and decreased acute care length of stay and promotes value based healthcare.

## 3. Purpose

The purpose of this quality improvement project was to implement an evidence-based standardized feeding approach, as recommended by the JCCHD-NPCQIC, for infants with single ventricle physiology with the goals of standardizing postoperative feeding practices, increasing interstage growth, reducing length of stay, and ultimately improving long-term neurodevelopmental outcomes in this vulnerable population. A standardized postoperative feeding protocol, which included the formation of an interprofessional feeding team, was initiated to reduce provider-dependent variability in feeding practices through translational science. The primary outcome objective in implementing a standardized evidence-based feeding approach was improved WAZ from home discharge following first-stage surgery until admission for second-stage palliation. The institutional review board waived this project as quality improvement.

Project questions included the following:

1. Do WAZ scores differ for infants with single ventricle physiology receiving a standardized feeding approach versus the standard of care at completion of the interstage?
2. Does caloric consumption at postoperative days 5–7 and 7–10 differ for infants with single ventricle physiology receiving a standardized feeding approach versus the standard of care following first-stage surgical palliation?
3. Do median lengths of stay following first- and second-stage surgical palliations differ for infants receiving a standardized feeding approach versus the standard of care?

## 4. Design and Methods

The quality improvement project was twofold: (1) formulate an evidence-based standardized feeding approach as an interprofessional congenital heart team and (2) implement the agreed upon protocol through a pediatric cardiac feeding team that would provide interstage continuity in both the inpatient and outpatient settings. An interprofessional feeding taskforce was formed with representation from pediatric cardiology, pediatric cardiothoracic surgery, pediatric critical care intensivists, neonatology, nursing leadership, a registered dietitian, speech therapist, and occupational therapist. The taskforce developed a mutually agreed upon evidence-based standardized feeding approach with inclusion criteria as follows:

- Hemodynamic stability with acceptable vital signs, reasonable urine output (≥2 mL/kg/hour), and good perfusion.
- Minimal or no acidosis, base deficit not worse than −4.
- Receiving no or low vasoactive support for at least 12 hours; may be on milrinone or epinephrine ≤0.03 mcg/kg/min.
- Infant's chest closed.
- Gestational age ≥35 weeks.
- Absence of congenital intestinal malformations or history of NEC.
- Absence of chylothorax.

The taskforce also established recommendations for nutrition initiation, rate of nutrition advancement, and required feeding team consultation. The pediatric cardiac feeding team was responsible for operationalizing the taskforce-developed protocol of a standardized approach to reduce healthcare provider variability.

The pediatric cardiac feeding team consisted of a registered dietitian, a speech therapist, an occupational or physical therapist, and a nurse practitioner. The feeding team rounded on infants who met inclusion criteria and made recommendations to the pediatric intensivists in the postoperative period during interprofessional rounds. The feeding team monitored nutritional status and growth throughout the interstage during hospitalization and later outpatient in collaboration with pediatric cardiology through the Single Ventricle Clinic.

Goals of the standardized feeding included optimizing calories through nutritional fortification. The primary nutrition goal was to obtain and maintain consumption of at least 120 calories per kilogram per day (Kcal/kg/d) by postoperative day (POD) 14 and throughout the interstage with expected weight gain of 20–30 grams per day (g/d). Staged goals of caloric consumption were set at 60 Kcal/kg/d by POD 3–5, 80 Kcal/kg/d by POD 5–7, and 100 Kcal/kg/d by POD 7–10.

Families of vulnerable children are important stakeholders in improving health outcomes. Parental engagement was secured through predischarge teaching by the feeding team and outpatient follow-up. Parents were educated on the goals for growth and equipped with home scales for daily weights and a binder in which to record the data. The nurse practitioner and pediatric cardiology team made weekly phone calls to parents ensuring nutrition and growth indicators were being achieved and nutritional adjustments were implemented. The infant was followed up closely through the Single Ventricle Clinic as recommended in the literature [13]. Data were collected on demographics, change in WAZ from discharge following first-stage palliation to admission for second-stage palliation, Kcal/kg/d, growth in g/d, and hospital lengths of stay for first-stage and second-stage surgical palliation.

The study utilized a cohort control group design. Participants were analyzed by the preintervention standard of care (control) condition (*n* = 5) or the intervention standardized interprofessional feeding approach condition (*n* = 5). The design has inherent bias in comparing outcomes over time but it is important to mention that no other major changes in standard of care were implemented concurrently. All data were entered and analyzed using IBM SPSS version 22. Mann-Whitney *U* tests were conducted to evaluate the expectation that infants in the intervention condition would consume more calories at 5–7 and 7–10 days postoperatively and have a higher WAZ and shorter length of stays following first and second surgeries, on average, than infants in the preintervention condition. The a priori alpha level was set at 0.10 for all statistical tests. A less conservative alpha was used due to the exploratory nature of the investigation.

## 5. Results

From September 2012 to September 2013, five infants received the intervention and were compared to five infants from the preceding year of September 2011 to August 2012, prior to process improvement.  Table 1 depicts the subject characteristics between groups.

**Figure 1:**
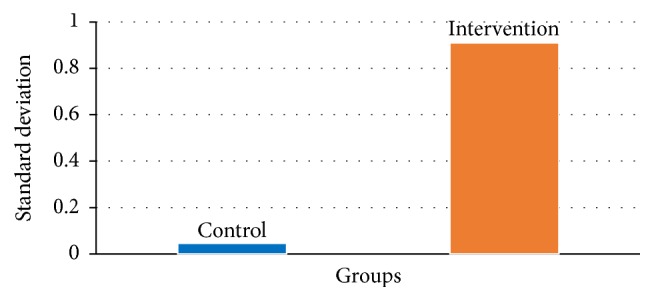
Change in the WAZ from discharge after first-stage palliation until admission for second-stage palliation.

Prior to a standardized feeding approach, the change in WAZ at admission for bidirectional Glenn was 0.05; following protocol implementation, the change in WAZ increased to 0.91, representing virtually a complete standard deviation improvement (see Figure 1). The results of the Mann-Whitney *U* test to evaluate the expectation that infants in the standardized feeding condition would have higher WAZ scores, on average, than infants in the preintervention condition were significant and in the expected direction, *z* = −1.78, *p* = 0.075, and *r* ^2^ = 0.32.

**Figure 2:**
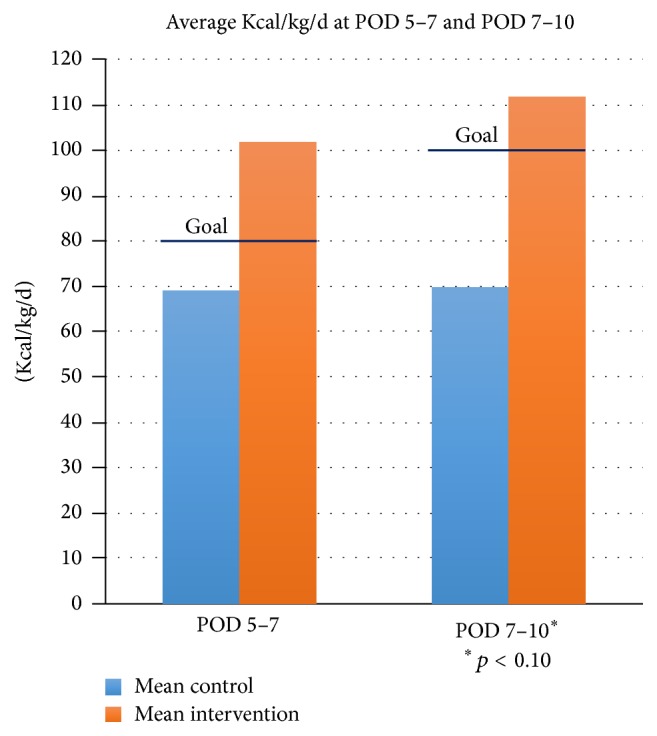
Mean caloric consumption on postoperative days 5–7 and 7–10. Goal caloric intake on POD 5–7 = 80 Kcal/kg/d; goal caloric intake on POD 7–10 = 100 Kcal/kg/d.

The results of the Mann-Whitney *U* test to assess differences after feeding at 5–7 days postoperatively were significant and in the expected direction, *z* = −2.40, *p* = 0.016, and *r* ^2^ = 0.58. Direct comparisons of caloric consumption found that the standardized feeding approach exceeded the goal of 80 Kcal/kg/d by postoperative days 5–7 at an average of 102 Kcal/kg/d, while the preintervention group demonstrated an average of 69 Kcal/kg/d (see Figure 2). Comparisons at 7–10 days postoperatively were also significant and in the expected direction, *z* = −1.77, *p* = 0.095, and *r* ^2^ = 0.31. The goal of 100 Kcal/kg/d for POD 7–10 was exceeded by the standardized feeding approach at 114 Kcal/kg/d, while the preintervention group demonstrated an average of 70 Kcal/kg/d. To assess equivalence, pretest comparisons were made between the preintervention and intervention cohorts on the dependent variables. No initial group differences were found across cohorts on the study variables.

**Figure 3:**
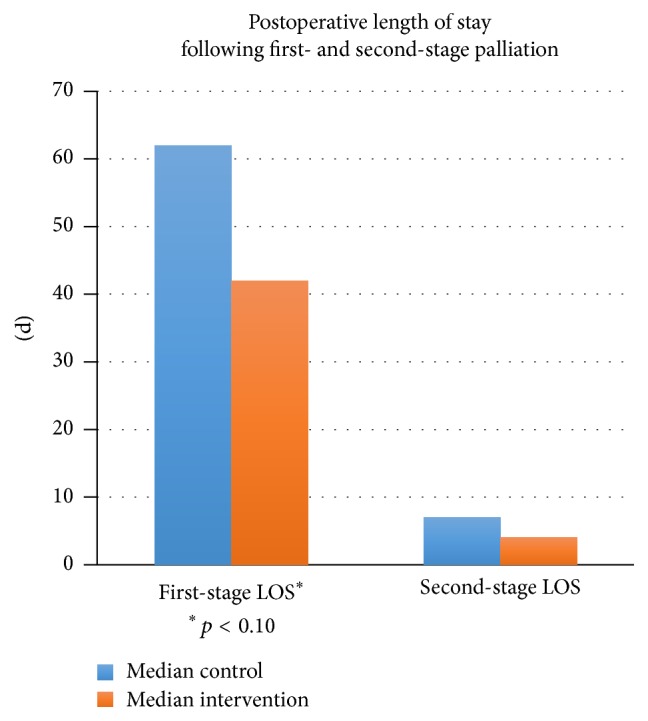
Median postoperative length of stay.

Median postoperative length of stay following first-stage palliation dropped 32% to 42 days (Figure 3). The results of the Mann-Whitney *U* test to evaluate the expectation that infants in the standardized feeding cohort would have a shorter length of stay following the first surgery, on average, than infants in the preintervention cohort were significant and in the expected direction, *z* = −1.68, *p* = 0.094, and *r* ^2^ = 0.28. The hospital length of stay for second-stage palliation decreased by 43% from 7 to 4 days. Statistical comparisons, however, did not reveal significant differences in length of stay following the second surgery (*p* = 0.195).

## 6. Limitations

The scarcity of infants with single ventricle physiology creates unique challenges in process improvement execution and evaluation. As such, a primary limitation is the difficulty in obtaining large sample sizes for rare health conditions such as single ventricle physiology. The small sample size in this intervention adversely influences statistical power to correctly identify significant differences between groups. A significant effect size was demonstrated but these results should be interpreted with caution due to the small sample and variables outside of the investigators control. In addition, a larger sample might provide data suitable for parametric statistical analysis.

## 7. Discussion

In this quality improvement project, infants following palliation for single ventricle physiology receiving a standardized feeding protocol were compared with a historical cohort. Both the intervention and the historical cohort included parent engagement with home weight monitoring to track growth indicators; only the intervention group received the benefit of the feeding team and the postoperative standardized feeding approach. Home weight monitoring has been associated with improved weight gain during the single ventricle interstage [14]. Infants receiving the standardized feeding approach demonstrated improved somatic growth and reduced length of stay, which is consistent with previous research. Decreasing length of stay has an impact on healthcare costs but, more importantly, the duration of ICU stay has been associated with poor neurocognitive outcomes [15]. In this cohort, standardized feeding led to improved nutrition and shorter duration of ICU stay, which has implications for long-term neurodevelopment in this vulnerable population. Higher WAZ indicates improved infant growth, which can be viewed as an indicator of adequate feeding and nutrition. This finding is similar to that reported by Anderson and colleagues [5] with improved WAZ by 0.98 associated with a standardized feeding approach during the single ventricle interstage. Improved caloric consumption results in improved growth as indicated by the change in WAZ during the interstage.

Cost savings are realized through decreased hospital length of stay following first-stage and second-stage surgical palliation as confirmed in the literature [12]. Median hospital length of stay decreased by 14–20 days following a standardized feeding approach for infants with single ventricle physiology (Figure 3). The average hospital cost for an infant in the NICU is $3500 per infant per day [16]. A cost saving of $490,000 to $700,000 per year in a ten-infant model could be realized following first-stage surgical palliation alone. Decreasing NICU length of stay decreases healthcare expenditures and supports value based medical care.

**Figure 4:**
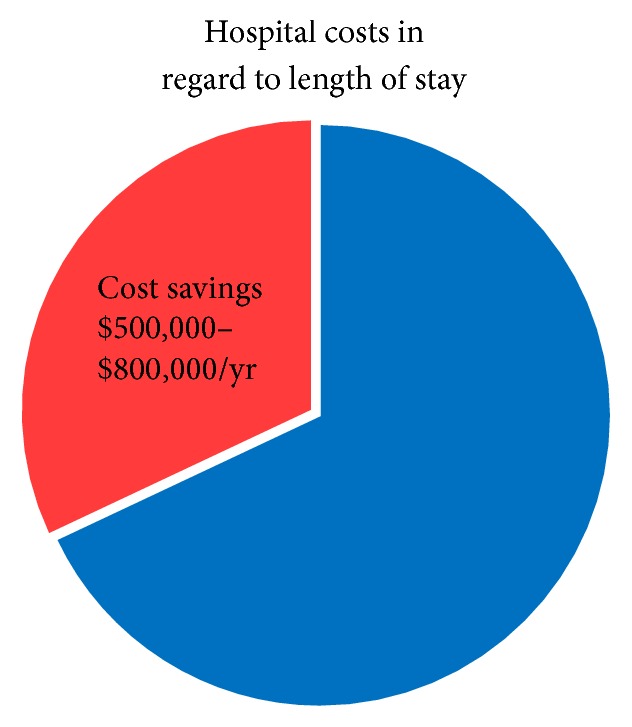
Potential cost savings per year for reduced length of stay in a 10-patient model.

In addition, the length of stay following second-stage palliation of the bidirectional Glenn was noted to decline from 7 to 4 days after implementation of a standardized feeding approach. This three-day decrease in hospital length of stay represents a potential cost saving of $40,000–$90,000 annually [17]. Since first- and second-stage palliative surgeries occur within the same year of life, this could represent an annual hospital saving of $500,000 to $800,000 per year if only ten infants with single ventricle physiology were treated (Figure 4). These costs do not reflect the probable decrease in hospital readmission rates due to complication prevention with improved nutritional status. In addition, these costs are calculated estimations based on average reported daily costs [16, 17]. Additional research is needed to determine whether actual hospital costs for infants following staged surgical palliation are congruent with the reported average daily expenditures.

The expenditure of adding a designated feeding team was not calculated because no additional full-time equivalent (FTE) positions were added for the project. Existing FTEs were simply reallocated for the intervention, optimizing efficiency. The addition of FTEs may be a financial consideration for other healthcare organizations.

## 8. Implications for Practice

The implementation of a standardized feeding approach which includes a feeding protocol and daily rounding by an interprofessional feeding team can demonstrate improved caloric intake and growth for infants with single ventricle physiology during the interstage. Due to the small sample size, the results should be viewed with caution; however, standardization in feeding practices yielded a positive change in weight-to-age *z*-scores, almost a full standard deviation with decreased hospital lengths of stay. Hospital length of stay following first-stage surgical palliation decreased over 30% and up to 40% following second-stage surgical palliation representing considerable healthcare expenditure savings following the implementation of this quality improvement project. The interprofessional cardiac feeding team reduced provider variability in feeding practices and increased compliance with the adopted feeding protocol. Goal caloric consumption can be achieved through an evidence-based feeding protocol and intervention of an interprofessional feeding team. The process of parental engagement through home weight monitoring and feeding education contributes to improved outcomes and strengthens the relationship of the family and healthcare providers [14]. Daily and colleagues found that parents of children with congenital heart disease strongly endorse interventions that may improve neurodevelopmental outcomes, such as better feeding practices [18]. Other vulnerable populations may benefit from standardization of feeding practices.

## 9. Conclusion

Translation science provides an opportunity to bring teams together and enhance professional collegiality while improving patient care outcomes. Stakeholders, including parents, serve as effective partners in process improvement. Parental engagement through home weight monitoring supports a standardized feeding approach to infants with single ventricle physiology during the interstage. Vulnerable patients benefit from a deliberate approach to clinical care through thoughtful collaboration in interprofessional teams as opposed to the inconsistency associated with random provider variability. Standardization in feeding practices for infants with single ventricle physiology during the interstage improves growth and nutrition while lowering hospital lengths of stay.

## Figures and Tables

**Table 1:** Subject characteristics.

Variable	Preintervention (*n* = 5)	Protocol (*n* = 5)
Gender: male	*n* = 3	*n* = 4
Birth weight (kg)	3.117 (2.84–4.04)	3.173 (2.7–3.63)
Gestational age	38	38
Physiology		
HLHS: AA/MA	1	1
HLHS: AA/MS	0	1
Unbalanced AVC	2	1
HRHS: TA	2	2

DOL at surgery	7.4 (3–15)	9 (7–16)^*∗*^
DOL at hospital DC	70.4 (44–101)	50 (30–69)
G-tube at DC	*n* = 2	*n* = 3
Change in WAZ	0.05	0.91^*∗*^
POD 5–7 Kcal/kg/d	69	102^*∗∗*^
POD 7–10 Kcal/kg/d	70	114^*∗*^
Median LOS, 1st stage	62	43^*∗*^
Median LOS, 2nd stage	7	4

^*∗*^ *p* < 0.10; ^*∗∗*^ *p* < 0.05.

HLHS: hypoplastic left heart syndrome; kg: kilogram; AA: aortic atresia; MA: mitral atresia; MS: mitral stenosis; AVC: atrioventricular canal defect; HRHS: hypoplastic right heart syndrome; TA: tricuspid atresia; DOL: day of life; DC: discharge; G-tube: gastric feeding tube; WAZ: weight-for-age *z*-score; POD: postoperative day; Kcal/kg/d: calories per kilogram per day; LOS: length of stay.
